# Supplementary material for: Predicting mortality risk for preterm infants using deep learning models with time-series vital sign data
Source: NPJ Digit Med. 2021 Jul 14;4:108. doi: 10.1038/s41746-021-00479-4 (PMC8280207; doi:10.1038/s41746-021-00479-4)
Supplement: Supplementary file 1 — Supplemental Material [file 41746_2021_479_MOESM1_ESM.pdf]

Supplementary Table 1: Patient Characteristics

	Survived (n=220)	Died (n=65)	Total cohort (n=285)
Birthweight, mean (SD), grams	1000 (261)	687 (202)	929 (281)
Gestational age, mean (SD), weeks	27.2 (2.2)	24.8 (1.7)	26.7 (2.3)
Male sex, n (%)	113 (51)	34 (52)	147 (52)
Race/ethnicity, n (%)			
White or Caucasian	118 (54)	35 (54)	153 (54)
Black or African American	92 (42)	24 (37)	116 (41)
Asian	2 (1)	1 (1)	3 (1)
Hispanic	2 (1)	2 (3)	4 (1)
Unknown/not reported	6 (2)	3 (5)	9 (3)
Age at admission, median (range), minutes	57 (12-463)	59 (21-278)	59 (12-463)
Length of stay, median (range), days	90 (40-383)	10 (1-387)	81 (1-387)
Multiple gestation, n (%)	39 (18)	17 (26)	56 (20)
Inborn	166 (75)	48 (73)	214 (75)
Final disposition, n (%)			
Discharged home			209 (73)
Transferred			11 (4)
Died			65 (23)

Supplementary Table 2: Cross-validation result of models

		Accuracy	Recall	Precision	Specificity	AUC
$n_h = 64$ $l_{highway} = 1$ $l_{cnn} = 1$ $p_d = 0.1$ $\beta = 1$	**fold1**	0.897	0.784	0.125	0.900	0.903
	**fold2**	0.840	0.853	0.071	0.839	0.909
	**fold3**	0.934	0.753	0.174	0.937	0.899
	**fold4**	0.883	0.729	0.154	0.887	0.877
	**mean**	0.888	0.780	0.131	0.891	0.897
$n_h = 128$ $l_{highway} = 1$ $l_{cnn} = 1$ $p_d = 0.1$ $\beta = 1$	**fold1**	0.900	0.734	0.123	0.904	0.912
	**fold2**	0.910	0.881	0.124	0.910	0.935
	**fold3**	0.913	0.631	0.119	0.918	0.866
	**fold4**	0.870	0.777	0.147	0.873	0.892
	**mean**	0.898	0.756	0.128	0.901	0.901
$n_h = 256$ $l_{highway} = 1$ $l_{cnn} = 1$ $p_d = 0.1$ $\beta = 1$	**fold1**	0.936	0.727	0.182	0.940	0.896
	**fold2**	0.901	0.946	0.121	0.900	0.966
	**fold3**	0.955	0.563	0.208	0.962	0.867
	**fold4**	0.882	0.659	0.142	0.888	0.830
	**mean**	0.918	0.724	0.163	0.923	0.890
$n_h = 512$ $l_{highway} = 1$ $l_{cnn} = 1$ $p_d = 0.1$ $\beta = 1$	**fold1**	0.929	0.693	0.160	0.933	0.883
	**fold2**	0.917	0.777	0.121	0.919	0.889
	**fold3**	0.958	0.597	0.227	0.964	0.868
	**fold4**	0.925	0.669	0.217	0.932	0.820
	**mean**	0.932	0.684	0.181	0.937	0.865
$n_h = 128$ $l_{highway} = 2$	**fold1**	0.954	0.490	0.193	0.962	0.859
	**fold2**	0.920	0.818	0.130	0.921	0.941
	**fold3**	0.935	0.656	0.161	0.940	0.893

$l_{cnn} = 1$	**fold4**	0.906	0.658	0.176	0.913	0.837
$p_d = 0.1$<br>$\beta = 1$	**mean**	0.929	0.656	0.165	0.934	0.883
$n_h = 128$	**fold1**	0.875	0.621	0.087	0.880	0.840
$l_{highway} = 3$	**fold2**	0.925	0.752	0.131	0.927	0.907
$l_{cnn} = 1$	**fold3**	0.949	0.749	0.216	0.952	0.908
$p_d = 0.1$	**fold4**	0.872	0.780	0.150	0.875	0.860
$\beta = 1$	**mean**	0.905	0.725	0.146	0.909	0.879
$n_h = 128$	**fold1**	0.907	0.832	0.143	0.909	0.924
$l_{highway} = 1$	**fold2**	0.824	0.770	0.060	0.825	0.864
$l_{cnn} = 2$	**fold3**	0.913	0.790	0.141	0.916	0.922
$p_d = 0.1$	**fold4**	0.861	0.688	0.127	0.866	0.835
$\beta = 1$	**mean**	0.877	0.770	0.118	0.879	0.886
$n_h = 128$	**fold1**	0.865	0.766	0.096	0.867	0.874
$l_{highway} = 1$	**fold2**	0.899	0.727	0.096	0.901	0.892
$l_{cnn} = 1$	**fold3**	0.881	0.619	0.087	0.886	0.852
$p_d = 0.05$	**fold4**	0.861	0.729	0.132	0.865	0.868
$\beta = 1$	**mean**	0.877	0.710	0.103	0.880	0.872
$n_h = 128$	**fold1**	0.936	0.714	0.180	0.940	0.926
$l_{highway} = 1$	**fold2**	0.912	0.759	0.113	0.914	0.869
$l_{cnn} = 1$	**fold3**	0.890	0.859	0.121	0.891	0.895
$p_d = 0.1$	**fold4**	0.856	0.653	0.117	0.862	0.823
$\beta = 2$	**mean**	0.898	0.746	0.133	0.902	0.878

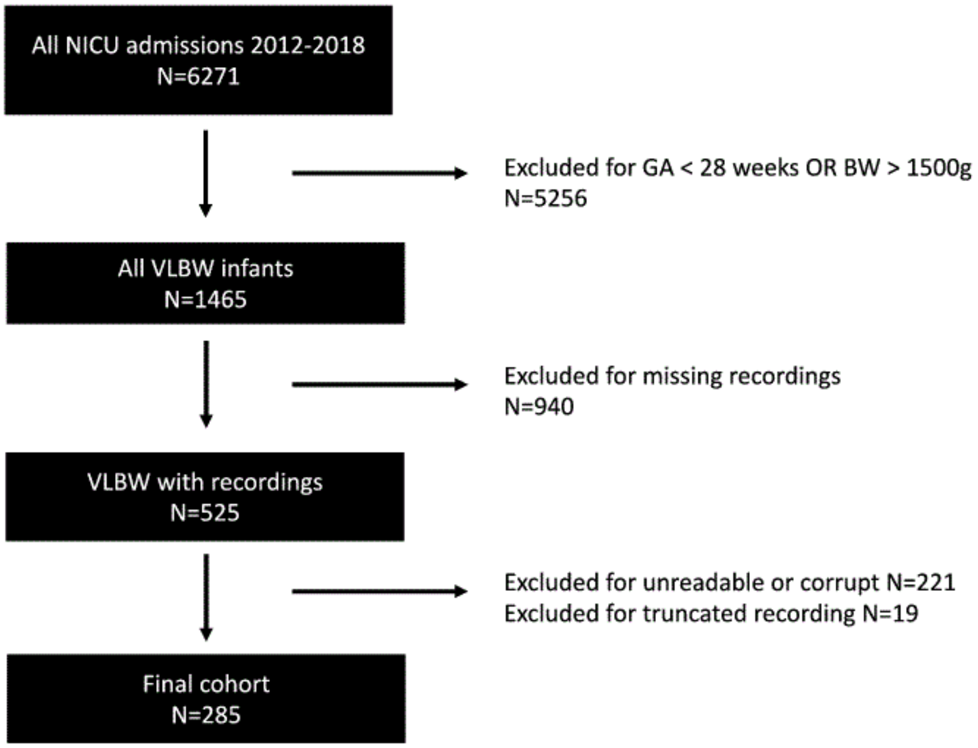

Supplemental Figure 1: Diagram demonstrating number of patients and reason for exclusion for development of final cohort.

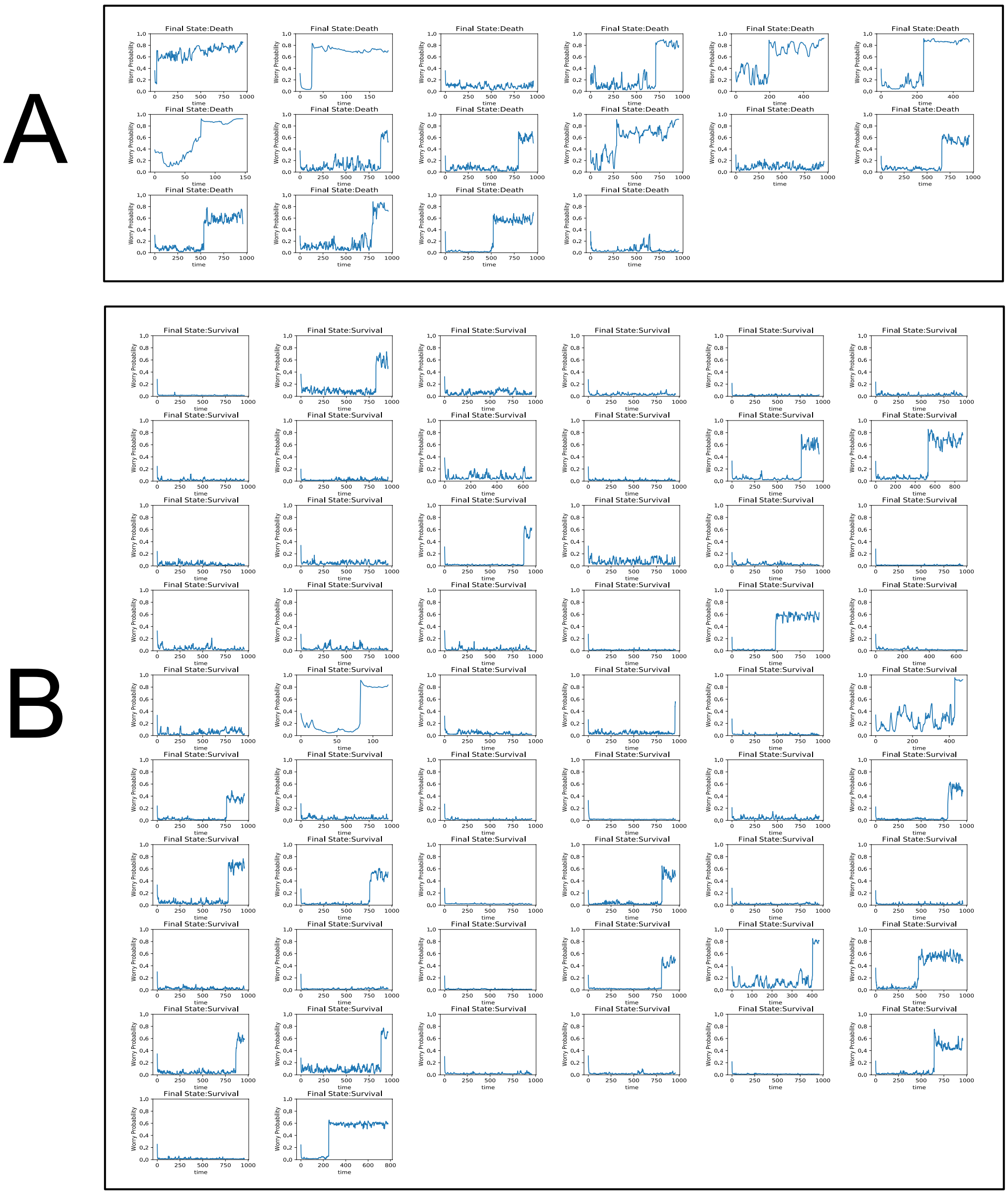

Supplementary Figure 2: Prediction results for each died (A) and survived (B) infant in first fold validation set

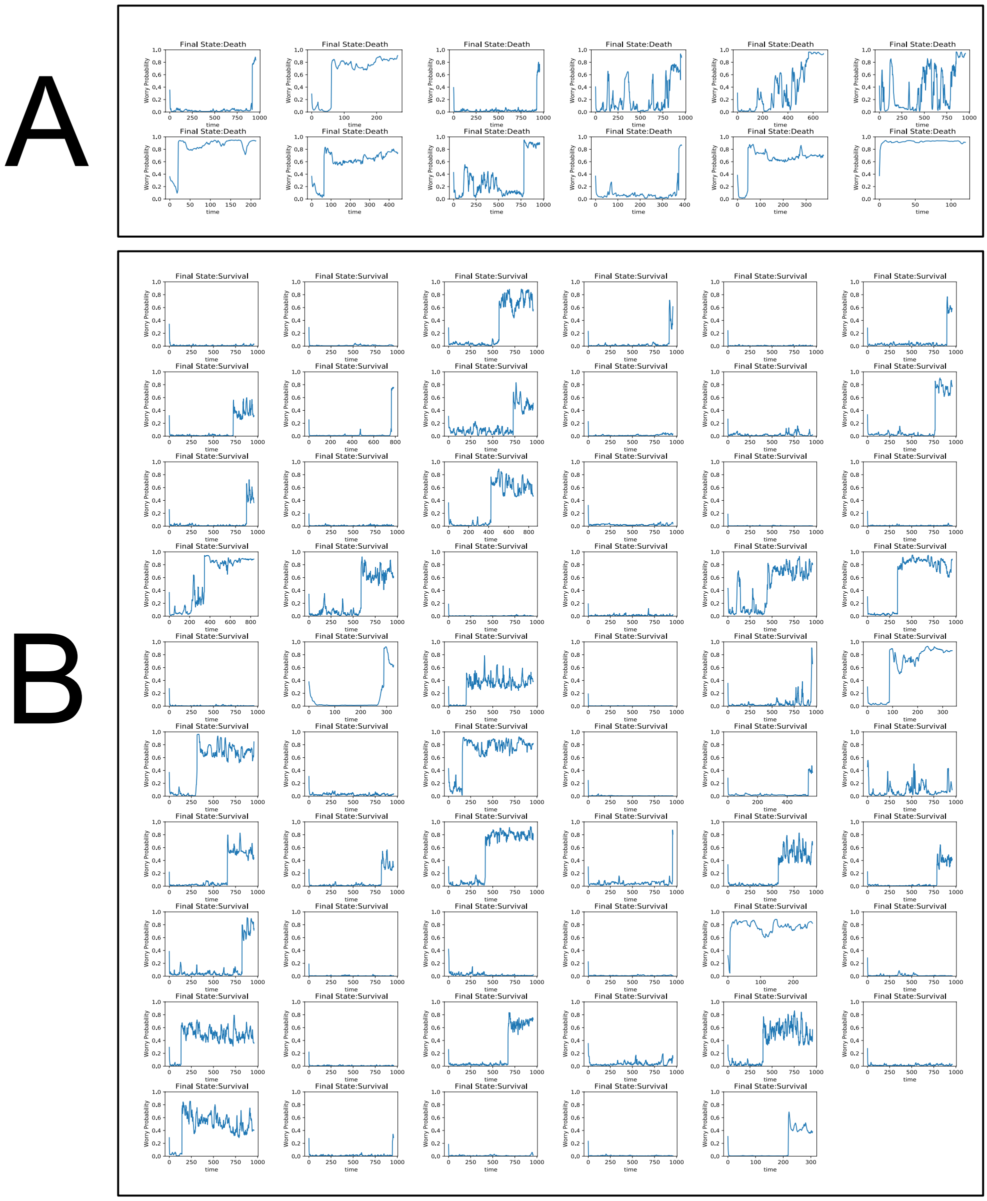

Supplementary Figure 3: Prediction results for each died (A) and survived(B) infant in second fold validation set.

A

B

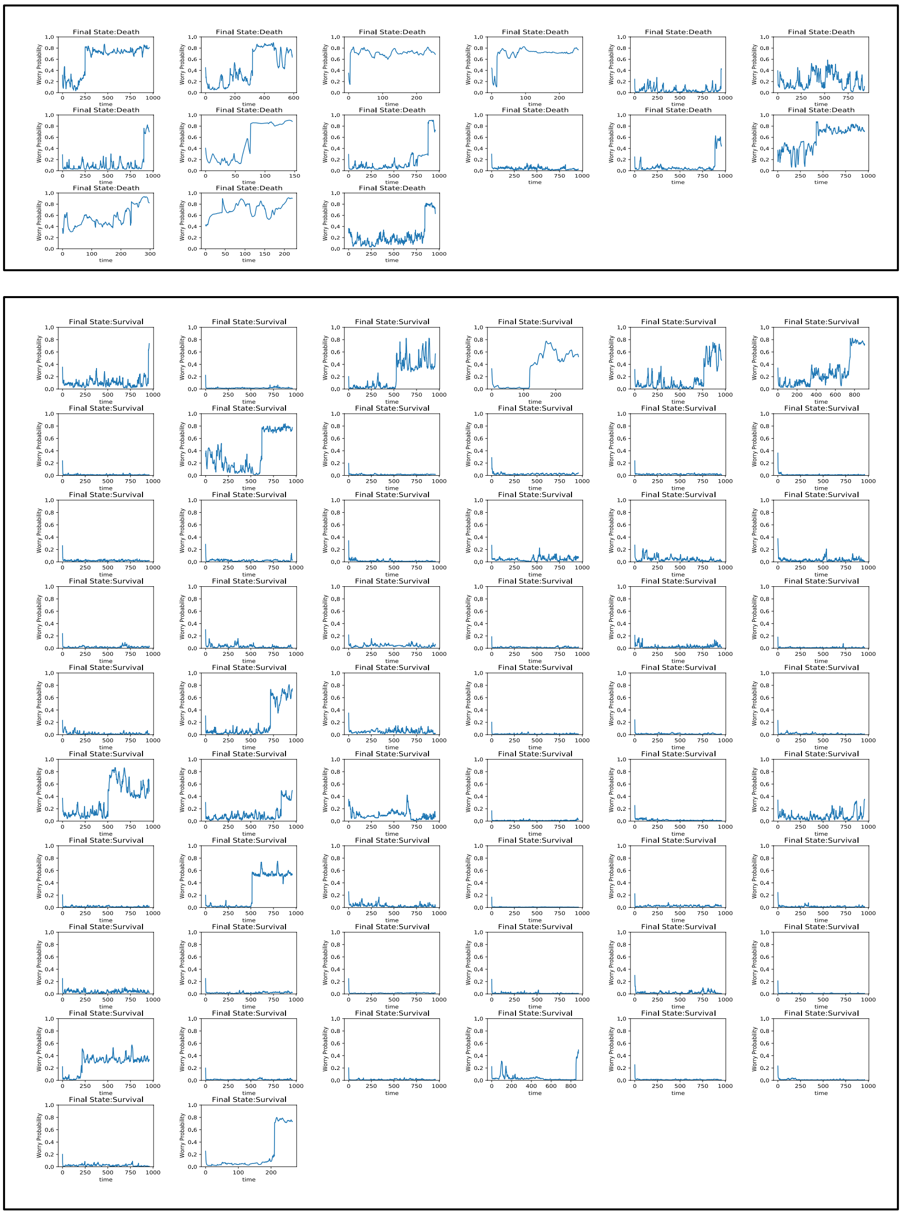

Supplementary Figure 4: Prediction results for each died (A) and survived (B) infant in third fold validation set.

A

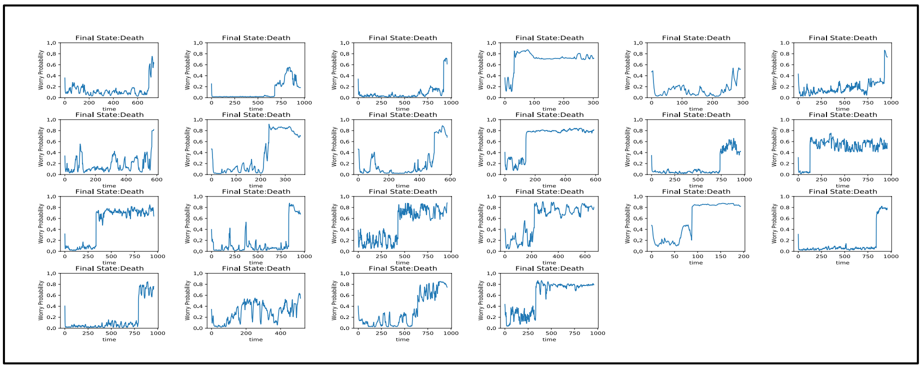

B

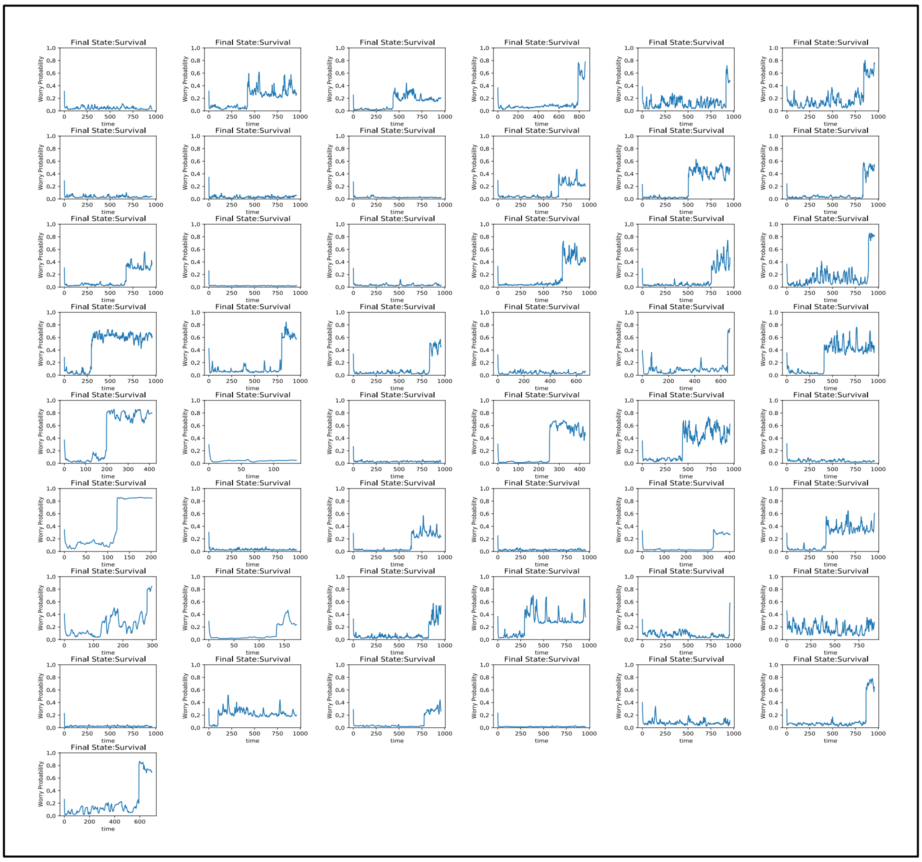

Supplementary Figure 5: Prediction results for each died (A) and survived (B) infant in fourth fold validation set.
