# Supplementary material for: Predicting mortality risk for preterm infants using deep learning models with time-series vital sign data
Source: NPJ Digit Med. 2021 Jul 14;4:108. doi: 10.1038/s41746-021-00479-4 (PMC8280207; doi:10.1038/s41746-021-00479-4)
Supplement: Supplementary file 2 — Reporting Summary [file 41746_2021_479_MOESM2_ESM.pdf]

nature research

Corresponding author(s): Vesoulis

Last updated by author(s): May 7, 2021

Reporting Summary

Nature Research wishes to improve the reproducibility of the work that we publish. This form provides structure for consistency and transparency in reporting. For further information on Nature Research policies, see our Editorial Policies and the Editorial Policy Checklist.

Statistics

For all statistical analyses, confirm that the following items are present in the figure legend, table legend, main text, or Methods section.

n/a	Confirmed	
☐	☒	The exact sample size (*n*) for each experimental group/condition, given as a discrete number and unit of measurement
☐	☒	A statement on whether measurements were taken from distinct samples or whether the same sample was measured repeatedly
☐	☒	The statistical test(s) used AND whether they are one- or two-sided<br>*Only common tests should be described solely by name; describe more complex techniques in the Methods section.*
☐	☒	A description of all covariates tested
☐	☒	A description of any assumptions or corrections, such as tests of normality and adjustment for multiple comparisons
☐	☒	A full description of the statistical parameters including central tendency (e.g. means) or other basic estimates (e.g. regression coefficient) AND variation (e.g. standard deviation) or associated estimates of uncertainty (e.g. confidence intervals)
☐	☒	For null hypothesis testing, the test statistic (e.g. *F*, *t*, *r*) with confidence intervals, effect sizes, degrees of freedom and *P* value noted<br>*Give P values as exact values whenever suitable.*
☒	☐	For Bayesian analysis, information on the choice of priors and Markov chain Monte Carlo settings
☒	☐	For hierarchical and complex designs, identification of the appropriate level for tests and full reporting of outcomes
☒	☐	Estimates of effect sizes (e.g. Cohen's *d*, Pearson's *r*), indicating how they were calculated

Our web collection on statistics for biologists contains articles on many of the points above.

Software and code

Policy information about availability of computer code

Data collection	Vital sign data were collected using the BedmasterEx software platform
Data analysis	The model was implemented in Python using Pytorch

For manuscripts utilizing custom algorithms or software that are central to the research but not yet described in published literature, software must be made available to editors and reviewers. We strongly encourage code deposition in a community repository (e.g. GitHub). See the Nature Research guidelines for submitting code & software for further information.

Data

Policy information about availability of data

All manuscripts must include a data availability statement. This statement should provide the following information, where applicable:

- Accession codes, unique identifiers, or web links for publicly available datasets
- A list of figures that have associated raw data
- A description of any restrictions on data availability

Privacy restrictions prevent publication of non-aggregated or raw clinical data.

Field-specific reporting

Please select the one below that is the best fit for your research. If you are not sure, read the appropriate sections before making your selection.

☒ Life sciences ☐ Behavioural & social sciences ☐ Ecological, evolutionary & environmental sciences

For a reference copy of the document with all sections, see nature.com/documents/nr-reporting-summary-flat.pdf

Life sciences study design

All studies must disclose on these points even when the disclosure is negative.

Sample size	All infants admitted to the NICU at St. Louis Children's Hospital, a level IV NICU serving urban, suburban, and rural populations have vital sign data prospectively archived into a research database (BedMaster EX, Excel Medical, Jupiter, FL). For this convenience sample of infants admitted between 2012 and 2018, we included all infants that were born prior to 32 completed weeks of gestation and had at least 6 hours of recorded vital sign data. Given the novel nature of this study, a priori sample size calculation was not performed. Only inborn and outborn preterm infants in their initial NICU hospitalization were included; infants with cyanotic heart disease, or those readmitted after hospital discharge are admitted to other units of the hospital and did not have any collected vital sign data (thus were not included).
Data exclusions	Infants were excluded due to missing, truncated, or corrupted recordings
Replication	5-fold cross validation was performed. Six different imputation techniques were evaluated.
Randomization	No randomization occurred
Blinding	No blinding was performed

Reporting for specific materials, systems and methods

We require information from authors about some types of materials, experimental systems and methods used in many studies. Here, indicate whether each material, system or method listed is relevant to your study. If you are not sure if a list item applies to your research, read the appropriate section before selecting a response.

Materials & experimental systems

n/a	Involved in the study	
☒	☐	Antibodies
☒	☐	Eukaryotic cell lines
☒	☐	Palaeontology and archaeology
☒	☐	Animals and other organisms
☐	☒	Human research participants
☐	☒	Clinical data
☒	☐	Dual use research of concern

Methods

n/a	Involved in the study	
☒	☐	ChIP-seq
☒	☐	Flow cytometry
☒	☐	MRI-based neuroimaging

Human research participants

Policy information about studies involving human research participants

Population characteristics	All infants < 32 weeks admitted to SLCH NICU 2012-2018 with at least 6 hours of valid vital sign recording were included. There were no other selection criteria, all eligible participants were included.
Recruitment	This was a retrospective study, no active recruitment was performed. Only infants who met all inclusion criteria were included.
Ethics oversight	The Washington University School of Medicine Institutional Review Board reviewed and approved this study under waiver of consent.

Note that full information on the approval of the study protocol must also be provided in the manuscript.

Clinical data

Policy information about clinical studies

All manuscripts should comply with the ICMJE guidelines for publication of clinical research and a completed CONSORT checklist must be included with all submissions.

Clinical trial registration	n/a

Study protocol	This not a clinical trial
Data collection	All infants < 32 weeks admitted to SLCH NICU 2012-2018 with at least 6 hours of valid vital sign recording were included
Outcomes	The primary outcome was death during initial NICU hospitalization, as noted in the clinical record
